# Non-Isothermal Crystallization Behavior of PEEK/Graphene Nanoplatelets Composites from Melt and Glass States

**DOI:** 10.3390/polym11010124

**Published:** 2019-01-12

**Authors:** Ángel Alvaredo, María Isabel Martín, Pere Castell, Roberto Guzmán de Villoria, Juan P. Fernández-Blázquez

**Affiliations:** 1 IMDEA Materials Institute, C/ Eric Kandel 2, 28906 Getafe, Madrid, Spain; angel.alvaredo@imdea.org (Á.A.); Roberto.Guzman@fidamc.es (R.G.d.V.); 2 FIDAMC, Foundation for the Research, Development and Application of Composite Materials, Avda. Rita Levi Montalcini 29, Tecnogetafe, 28906 Getafe, Madrid, Spain; Maria-Isabel.Martin@fidamc.es; 3 Fundación AITIIP, Pol. Ind. Empresarium, C/ Romero 12, 50720 Zaragoza, Spain; pere.castell@aitiip.com

**Keywords:** graphene, PEEK, WAXS, SAXS, synchrotron, cold crystallization, dynamic crystallization, multifunctional composite materials

## Abstract

The effect of the graphene nanoplateletets (GNP), at concentration of 1, 5 and 10 wt %, in Poly ether ether ketone (PEEK) composite crystallization from melt and during cold crystallization were investigated by differential scanning calorimetry (DSC) and real time X-ray diffraction experiments. DSC results revealed a double effect of GNP: (a) nucleating effect crystallization from melt started at higher temperatures and (b) longer global crystallization time due to the restriction in the polymer chain mobility. This hindered mobility were proved by rheological behavior of nanocomposites, because to the increase of complex viscosity, G′, G″ with the GNP content, as well as the non-Newtonian behavior found in composites with high GNP content. Finally, real time wide and small angle synchrotron X-ray radiation (WAXS/SAXS) X-ray measurements showed that GNP has not affected the orthorhombic phase of PEEK nor the evolution of the crystal phase during the crystallization processes. However, the correlation length of the crystal obtained by WAXS and the long period (L) by SAXS varied depending on the GNP content.

## 1. Introduction

Poly ether ether ketone (PEEK) is a well-known high performance thermoplastic polymer with attractive properties, such as high thermal stability and high melting temperature [1,2,3]. PEEK is semicrystalline and its physical and mechanical properties depend on the crystalline morphology as well as the degree of crystallinity, both highly sensitive to processing conditions and additives [3,4].

The importance of PEEK in the industry is on the rise, with a large and growing number of applications in different industrial sectors. One of the main handicaps for PEEK applications is its processing, where the crystallization plays an important role. For that reason, the crystallization process has been widely studied by different techniques, such as differential scanning calorimetry (DSC), temperature modulated differential scanning calorimetry (TMDSC), dielectric relaxation analysis, time-resolved synchrotron X-ray diffraction, and electron microscopy [5,6,7,8,9,10]. It is well-known that the crystallization kinetics of polymers involves two competitive processes, nucleation and growth [11]. However, in the case of PEEK, this mechanism is more complex, because both processes, nucleation and growth, happen in two stages [12]. The first one corresponds to the primary crystallization, the crystalline lamellae arranges into spherulites. The second process is a secondary crystallization that takes place in the inter-lamellar spherulitic region [5,12]. This crystallization behaviour is also associated to the melting process, due to the existence a double melting peak [13,14,15]. Two main reasons explain this fact: the former is the melting-recrystallization-melting process, common semicrystalline polymers. The latter, the existence of two different crystalline morphologies due to the complex crystallization process, as was concluded by the TMDSC results [9].

Traditionally, the kinetics and crystalline structure has been studied in polymers through isothermal crystallization conditions [16]. However, the study of non-isothermal crystallization from molten state has an important technological relevance, because it is much closer to standard industrial processing conditions [17]. Therefore, the knowledge of non-isothermal crystallization process may help in industrial process optimization and then the quality of the future PEEK products [16]. Nevertheless, there are few works about the crystallization and morphology of neat PEEK during non-isothermal test so far [16,18]. These studies observed also the two competing nucleation and growth crystallization processes [16,18].

Furthermore, the crystallization of PEEK can be modified by the addition of nanofillers [5]. It is reported that the addition of nanofillers to PEEK can have an influence in its crystallization process, such as in the nucleation as in the crystal growth [3,19]. During the nucleation, nanofiller may act as nucleating agent promoting heterogeneous nucleation, and then increasing the crystallization temperature (*T*_c_), crystallization rate, and degree of crystallinity in some cases [5,19,20]. During the growth of crystallites, nanofillers could restrict the mobility of the polymer chains, reducing the crystallization growth rate, crystallite size, and the level of crystallinity [3,5,19,21]. Graphene nanoplatelets (GNP), which are formed of several layers of graphene, have attracted the attention of the scientific (academic and industrial) community, because they have good balance between some superb properties of graphene and their ease and low cost production when compared to pristine graphene [22]. The effect of GNP in the crystallization of PEEK is still unclear due to the few studies that are available in the literature. A slight increase in crystallinity of PEEK and a nucleation effect during cold crystallization have been reported with the addition of graphene oxide (GO) and GNP [23,24]. In contrast, a decrease in the crystallization temperature of PEEK with the addition of 1 wt % of GO is also reported [25]. This controversy is also found in other nano-carbons, i.e., carbon nanotubes (CNTs) [19,21,26,27]. Rong et al. [19] observed higher crystallization temperature with the addition of low contents of CNT’s, such if they were functionalized with carboxylic acid or not. Nevertheless, the acid functionalization of CNT’s reduced the *T*_c_ for high f-CNT’s content (5 wt %), due to mobility reduction of PEEK chains. On the other hand, Diez-Pascual et al. [21] observed this *T*_c_ decrease, even at very low contents of CNT´s (0.1 wt %).

Therefore, the study of opposite effect of carbon-based nanofillers in the PEEK crystallization behaviour, as accelerating due to their nucleating effect or as hindering due to the restriction mobility of PEEK chains, is the main aim in this work. For that purpose, PEEK and PEEK/GNP composites (1, 5, 10 wt %) were obtained by extrusion moulding, followed by hot press processing. Crystallization kinetic was studied by the DSC technique. In situ non-isothermal crystallization experiments were followed by wide and small angle synchrotron X-ray radiation (WAXS and SAXS) to analyse the crystalline morphology. These non-isothermal crystallization studies were carried out from glass and from melt states, which allow for comparing the crystallization processes at quite different temperatures. Furthermore, the rheological properties of PEEK and PEEK/GNP nanocomposites were analysed to evaluate the effect of GNP within the PEEK matrix, mainly in the PEEK chain mobility.

## 2. Materials and Methods

### 2.1. Materials

The PEEK matrix (PEEK-90G) was provided in pellets by Victrex plc, Lancashire, UK. This grade of PEEK presents the following physical characteristics: Glass transition temperature (*T*_g_) = 143 °C, melting temperature (*T*_m_) = 343 °C, density (*d*_25°C_) = 1.3 g/cm^3^, and viscosity (η_400°C_) = 90 Pa·s. Graphene Nanoplatelets (GNP, avanGraphene 1–2 layers) were purchased from Avanzare (Navarrete, La Rioja, Spain).

### 2.2. Nanocomposite Films Preparation by Extrusion-Moulding and Hot Press

Melt-blending of PEEK nanocomposites was performed in an industrial extrusion-compounding machine (Coperion ZSK 26, Stuttgart, Germany) that was equipped with a 26 mm diameter co-rotating twin-screw and with two Brabender gravimetric feeders. The temperature profile of the extruder was set at 330 °C at the feeder increasing to 360 °C at the nozzle. A specifically designed, high shear rate screw profile was used to ensure proper dispersion. A rotor speed of 250 rpm was used to process all of the nanocomposites. The dosage of PEEK was set at 10 kg/h and different quantities of GNP were dosed in a lateral feeder to achieve a final concentration in the nanocomposites of 1, 5, and 10 wt % raw GNP. The molten material was extruded through a 2 mm diameter die at a constant output rate to give the different compositions. The extrudate strand was quenched immediately in a water bath at room temperature, dried, and cut into small pellets. Pure PEEK material was processed under the same conditions as a reference material.

After that, around 5 g of nanocomposite or neat PEEK were used to produce films, which an approximate thickness of 0.1 mm, using a stainless steel frame to control film dimensions by holding resin flow. Films with thickness of 1 mm were produced for rheological experiments. Aluminium plates were placed on the bottom and top surfaces of the hot-press plates. In addition, Kapton film was used between the aluminium plates and PEEK/GNP pellets in order to avoid the adhesion between them. The films were made using a hot-press at 380 °C. Firstly, for five minutes no pressure was applied and then 20 bars during 5 min were applied. Two different cooling processes were carried out. Films were cooled in air at 2 °C/min to obtain semicrystalline films. Films were also quenched in water to quickly reduce the temperature below the glass transition temperature with the aim to produce amorphous films.

### 2.3. Rheological Measurements

Dynamic shear tests were done to study the role of GNP on the rheological properties of PEEK. Films with 1 mm thickness were cut in circles with 25 mm diameter. Rheological measurements were carried out using a rheometer model AR-G2 from TA Instruments (New Castle, DE, USA) with aluminium disposable plates of 25 mm in diameter under air atmosphere. The gap was maintained as close as possible to 1 mm in all of the experiments. Dynamic strain sweeps were carried out to calculate the linear viscoelastic region. After that, one dynamic frequency sweep per sample was performed at 380 °C, using a frequency range from 0.1 to 100 Hz and a strain of 1%.

### 2.4. Differential Scanning Calorimetry

The effects of graphene nanoplatelets (GNP) on the non-isothermal crystallization behaviour were carried out by a calorimeter (TA Instruments, model Q200), through two types of test: melt crystallization and cold crystallization, using 5–10 mg of each film. Melt crystallization: the samples were heated to 400 °C at a rate of 10 °C/min and then held isothermally for 5 min for melt completely. Afterwards, these samples were cooled from 400 to 20 °C at different cooling rates of 2.5, 5, 10, 20 °C/min (Figure S1). Subsequently, samples were heated to 400 °C at a heating rate of 10 °C/min. Cold crystallization: in this case, it was necessary to prepare a new aluminium pan for each test. The amorphous samples were heated to 400 °C at rates of 2.5, 5, 10, 15, and 20 °C/min (Figure S2).

The degree of crystallinity of samples crystallized from melt (*X*_c_), and in samples crystallized from glass (*X*_cc_) was calculated according to [28]:

(1) $$X_{c}=\frac{\Delta H_{m}}{\left(1-\phi \right)\cdot \Delta H_{0}}$$

where Δ*H*_0_ is the heat of fusion of 100% crystalline PEEK, taken as 130 J/g [29], ϕ is the weight fraction per unit mass of nanoreinforcement and Δ*H*_m_ is the heat of melting calculated by the integration of the melting peak for all samples. The melting enthalpy minus cold crystallization enthalpy (Δ*H*_m_ − Δ*H*_cc_) in a heating ramp of quenched samples is associated to crystallinity obtained during quenching processes. It was calculated by integration from 146 (previous cold crystallization) to 375 °C (after melting). Crystallization peaks were the peak minimum position and crystallization times were calculated from the onset of crystallization to the offset.

### 2.5. Wide and Small Angle X-ray Diffraction

Synchrotron X-ray diffraction was used to record simultaneously wide- and small-angle scattering (WAXS and SAXS, respectively) patterns during a non-isothermal crystallization experiment at typical DSC cooling or heating rates. The experiments were carried out at the Non Crystalline Diffraction (NCD) beamline within ALBA synchrotron light facility (Cerdanyola del Vallés, Barcelona, Spain). The wavelength was 1 Å, delivering a high photon flux onto the sample. SAXS and WAXS patterns, which were obtained every 2 °C during melting and cooling cycles at 10 °C/min using a Linkam controller, were obtained through CCD detectors provided by ADSC (Quantum 210r) and Rayonix LLC (LX255-HS), with an active area 210 × 210 and 85 × 255 mm^2^, respectively. The integration profile was taking using the DAWN software [30].

The Bragg’s long period was calculated through the position of the scattering maximum (*q*_max_) from the Lorentz-corrected SAXS profile [31], following the next expression:

(2) $$L_{B}=\frac{2\cdot \pi }{q_{\max}}$$

where *q* = (4π/λ) sin ϴ is the scattering vector and 2ϴ is the scattering angle. Bragg´s long period is the average periodicity of the lamellar stack, which corresponds to the sum of the average thickness of the crystal lamellae and of the interlamellar amorphous regions [6].

## 3. Results and Discussion

### 3.1. Rheological Properties

**Figure 1:**
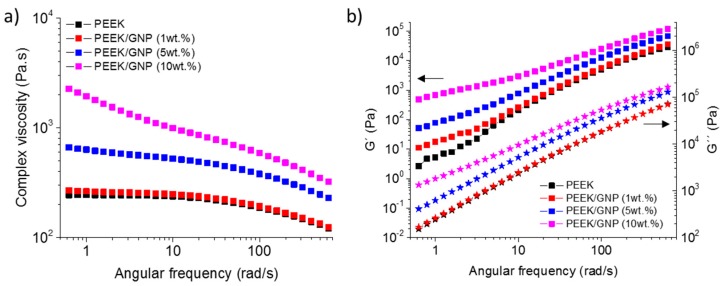
(**a**) Complex viscosity (η*) and (**b**) square symbols for storage modulus (G′) and star symbols for loss modulus (G″) obtained from dynamic frequency sweep.

The role of GNP on the viscoelastic behaviour of PEEK was studied from the dynamic frequency sweep measurements. Complex viscosity, storage modulus, and loss modulus for neat PEEK and the nanocomposites are shown in Figure 1. Pure PEEK showed the typical behaviour of thermoplastic, Newtonian response in the complex viscosity at a low frequency [32]. At this low frequency, the elastic modulus, G′, followed the expected behaviour when polymer chains are fully relaxed, and then they presented initial stages of polymer-like terminal flow behaviour. The addition of 1 wt % of GNP had a limited effect. This composite kept the Newtonian behaviour, but at low frequencies the complex viscosity began to be slightly higher. More differences were found in G′, where the terminal flow behaviour started to be hindered, showing the beginning of rubbery plateau formation at low frequencies. G′ at high frequency was practically unaffected due to a strong shear thinning behaviour [33], and G″ was similar in the whole frequency range, and consequently not important restrictions were expected in the polymer chain mobility. However, the increase of GNP content to 5 and 10 wt % triggered severe changes in the rheological behaviour of composites. Firstly, complex viscosity, G′, and G″ rose in the whole frequency range analysed. In the case of 5 wt %, at low frequency, the complex behaviour began to leave the Newtonian behaviour and G′ continued the progressive evolution to form a rubbery plateau, due to the increase of the polymer chain mobility restrictions. This trend was more pronounced for composite with 10 wt % of GNP, the Newtonian behaviour disappeared, and in G′, the rubbery plateau formation was unmistakable. This fact is due to the transition from liquid-like to solid-like viscoelastic behaviour, caused by the interactions particle-particle that dominate over the polymer-filler interactions, forming an incipient interconnected network of GNP [34,35]. This network limits the large-scale motions of PEEK chains, affecting the global crystallization process, as is discussed below.

### 3.2. Thermal Behaviour of PEEK Nanocomposites

**Figure 2:**
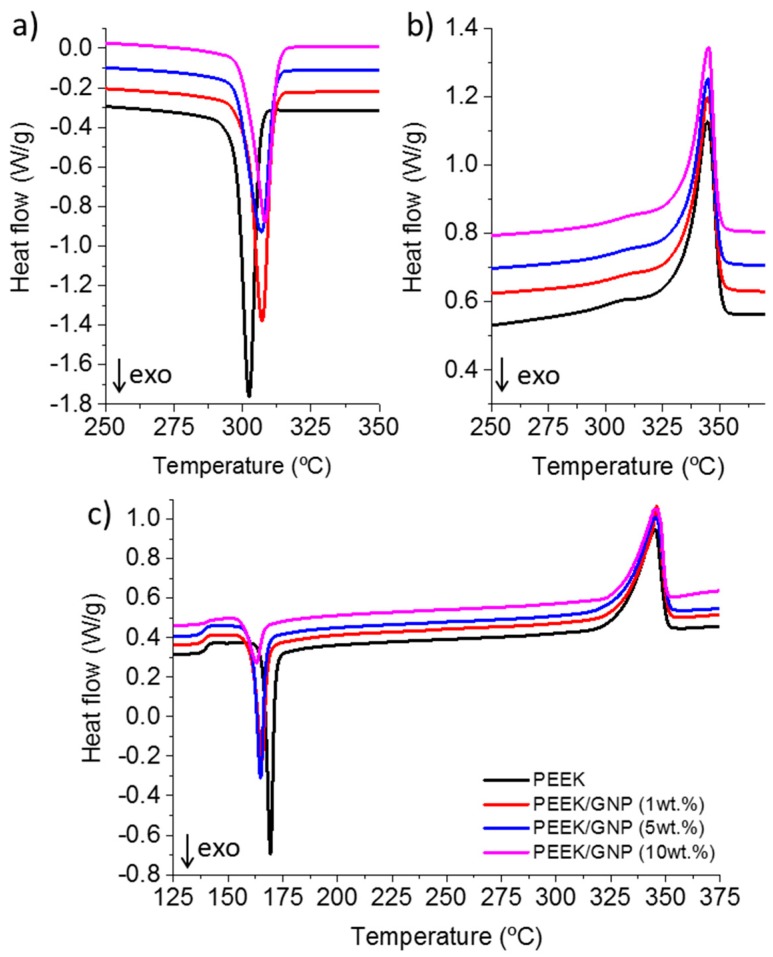
**Figure 2**. Differential scanning calorimetry (DSC) thermographs of poly ether ether ketone (PEEK) and PEEK with 1, 5, and 10 wt % of graphene nanoplateletets (GNP) during non-isothermal tests, (**a**) crystallization from melt, (**b**) subsequent melting, and (**c**) heating of quenched samples.

The DSC curves during cooling from melt, subsequent heating, as well as heating from glass state for PEEK and PEEK/GNP nanocomposites and are plotted in Figure 2. All of the ramps were performed at 10 °C/min. Figure 2a shows the cooling from molten state, where the influence of GNP is evident, because all the composites began the crystallization at higher temperature without important differences among them. Likewise, a widening of the exothermic process was clearly observed and corroborated by the full width at half maximum values (FWHM), as is discussed below. However, the melting curves were similar in all the cases indicating that the melting behaviour of PEEK was not modified by the addition of GNP after a non-isothermal crystallization at 10 °C/min.

To discuss in detail these observations, Table 1 summarizes the values of temperatures for the beginning of crystallization (*T*_onset_), temperature of the exothermic peak position (*T*_c_), FWHM, crystallization times calculated from *T*_onset_ to *T*_offset_ (*t*_c_), melting temperature (*T*_m_), and melting enthalpy (Δ*H*_m_). GNP acted as nucleant of PEEK, crystallization of the composite with just 1 wt % of GNP increased its *T*_c_ and *T*_onset_ in around 5 °C, composites with higher GNP content crystallized at similar temperatures and an increase of only 1.5 °C was observed in *T*_onset_ or *T*_c_, even with a GNP concentration of tenfold more. However, the extent of the crystallization process was longer with the content of GNP, with this increase being more important for samples with high concentration of GNP (5 and 10 wt %). In the case of crystallization from glassy state (Figure 2c), similar behaviour was observed. Composites showed a decrease of *T*_onset_ and *T*_cc_ (cold crystallization temperature) due to GNP nucleating behaviour and crystallization process durations were longer as well. Nevertheless, we have to consider that the cold crystallization process could be affected if the crystallization had already begun during the quenching process. This fact was analysed by the Δ*H*_m_ − Δ*H*_cc_ (melting enthalpy minus cold crystallization enthalpy). Table 1 shows these results, where pure PEEK and composites with 1 and 5 wt % of GNP content could be considered to be amorphous. However, composite with 10 wt % presented an enthalpy of 15 J/g, resulting in 10% of crystallinity, and then this nanocomposite did not crystallize from pure glassy state. This fact will be discussed in the synchrotron result section.

On the other hand, the melting enthalpy values showed no significant changes with the addition of GNP and the degree of crystallization remained constant above 40% in the case of the samples that were crystallized from melt. The same unaltered crystallinity with the addition of graphene or CNT´s has been previously reported [5,21,25]. However, a slightly increase in the degree of crystallinity was reported after cold crystallization in quenched the sample with the addition of 2 wt % of GNP [23]. In our case, the nanocomposites with 5 wt % also showed a slight increase in the crystallinity, but a higher concentration of GNP (10 wt %) has the opposite behaviour. It should be noticed that this last sample was not totally amorphous, therefore the cold crystallization in this case was not from pure glass, it might affect the final crystallinity that is reached.

**Figure 3:**
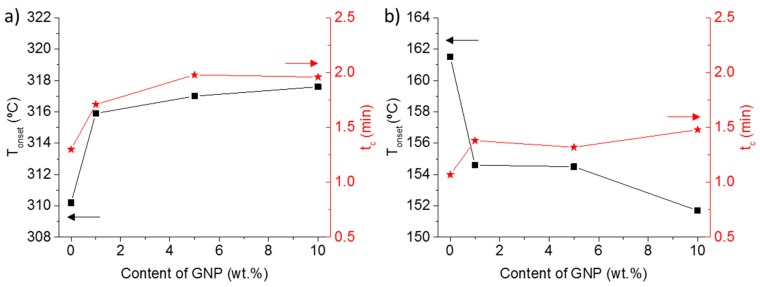
Onset Crystallization temperature (square symbols) and crystallization time (star symbols) against the content of GNP. Samples crystallized from melt (**a**) and from glass (**b**).

Summarizing, GNP used in our composites has two main effects in the crystallization process. First, the increase of *T*_c_ in the nanocomposites during the crystallization from melt, the lower *T*_cc_ during the crystallization from glass, as well as the inability for reaching a total quenching in the composite with 10 wt %, evidenced the nucleating effect of GNP in PEEK. Second, crystallization times showed an increase with the addition of GNP. As it was commented previously, the addition of GNP limited the large-scale motions of polymer chains, increasing the complex viscosity as compared to neat PEEK. Therefore, the molecular mobility of the PEEK chains was reduced, hindering the crystallization and increasing the global crystallization time, which is probably due to a reduction of the crystallization growth rate [5]. Figure 3 shows this double effect when GNP was added to PEEK: (a) nucleation. The crystallization started at higher temperatures in crystallization from melt and lower temperature from glass. (b) GNP restricts the PEEK chain mobility. The global crystallization time was longer. This factor justifies the results that were observed in the literature where composite made by bi/tri layer graphene in PEEK at a concentration of 2 and 5 wt % reduced the *T*_c_ few degrees [23]. This double effect of the nanocarbons in the global crystallization rate of PEEK has been observed also in CNTs, independently of they were functionalized or not. Lower concentration of CNT increased the *T*_c_, but the concentration of 5 wt % hindered the crystallization process due to the restriction in the polymer mobility [19].

#### Crystallization Kinetics

Ozawa equation describes the crystallization of polymers during non-isothermal conditions [36], taking into account the effect of cooling or heating rate (β).

(3) Xc(T)=1−e(K(T)βm)

(4) $$\log\left[-\ln(1-X_{c}(T)\right]=\log K\left(T\right)-m\cdot \log\beta$$

where *Χ*_c_(*T*) is the relative degree of crystallization at temperature *T*, *K*(*T*) is a cooling function and it describes crystallization rate, which depends only on temperature, and m is defined as the Ozawa exponent depending on the dimensions of the crystal growth [3]. The Ozawa´s method is valid only for polymers that log[−ln(1 − *X*_c_(*T*))] against log(β) becomes linear, being the values of *K*(*T*) and m derived from the intercepts and the slopes of the curve [1,3]. Previous to applying the Ozawa equations, the degree of crystallinity against the time was calculated and plotted for all of the samples, as shown in Figures S3 and S4. Both figures reveal a sigmoidal shape and a curvature in the upper part of the curves, which can be attributed to the secondary crystallization of PEEK [13,37].

The crystallization behaviours from melt (Figure S5) and glass states (Figure S6) of the neat PEEK and the composites of PEEK with GNP were not linear. Therefore, the Ozawa model failed in the range of crystallization rates that were used in this study. The failure of the Ozawa method for a similar range of rates to describe the crystallization behaviour of PEEK has been reported previously [1,3,38], and the main reason is that this model does not take into account the secondary crystallization and/or heterogeneous nucleation, which have been observed in our experiments.

Another common crystallization kinetic model to describe the crystallization behaviour of polymers during non-isothermal conditions is the Avrami analysis modified by Jeziorny [3,37,38,39]. In the Avrami equation as compared to Ozawa equation, the cooling rate is replaced by the time [3,37]:

(5) $$X_{c}\left(t\right)=1-e^{\left(-Z_{t}\cdot t^{n}\right)}$$

(6) $$\log\left[-\ln(1-X_{c}(t)\right]=\log\;Z_{t}+n\cdot \log\;t$$

being *Χ*_c_(t), the relative degree of crystallinity at time t, *Z*_c_, the crystallization rate, which is temperature dependent and n, Avrami index [3,37]. For non-isothermal conditions, it is necessary to modify the Avrami equation replacing the value of *Z*_t_ for *Z*_c_, as follow [3,37]:

(7) $$\log\;Z_{c}=\frac{\log Z_{t}}{\beta }$$

**Figure 4:**
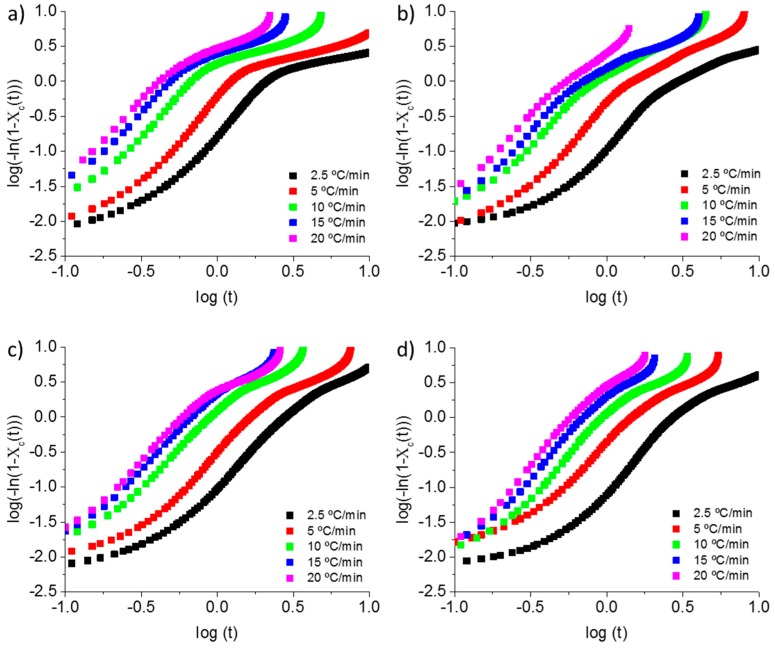
Modified Avrami plots at various cooling rates. (**a**) neat PEEK, (**b**) PEEK/GNP (1 wt %) (**c**) PEEK/GNP (5 wt %), and (**d**) PEEK/GNP (10 wt %).

As shown in Figure 4 and Figure S7, the curve of log(−ln(1 − *X*_c_(t))) against log t presents a linear trend between 3 to 50% of *X*_c_(t). Therefore, modified Avrami equation can be applied in the first stage of the crystallization. Avrami constants, *n* and *Z*_t_ can be determined from the slope and intercept of the plot, respectively. Both are summarized in Table 2. The n value is found between 2.2 to 2.4 in all samples that were crystallized from melt (Table 2). No significant changes can be observed while adding GNP and all of the samples showed two-dimensional growth habit [40]. Similar behaviour was observed in polyphenylene sulphide with the addition of GNP [41] and in PEEK matrix adding CNT´s [19]. The *n* values of all composites and neat PEEK are higher in the crystallization from glass than in crystallization from melt, suggesting that the type of nucleation and growth of the primary nucleation is different from the kind of non-isothermal crystallization from melt or from cold crystallization. This behaviour was previously reported in PEEKK and PEKEKK [2,38]. Moreover, the *n* values of neat PEEK during crystallization from glass state are higher than the n values of nanocomposites (Table 2). The different n value between the samples showed a change in growth habit adding GNP for cold crystallization. An index close to 4 indicates a three-dimension growth of PEEK crystallites, while a index between 2 to 3 means two-dimensional growth [40].

On the other hand, *Z*_c_ values showed a clear trend, decreasing when adding higher percentages of GNP. The lower *Z*_c_ values of PEEK/GNP samples is probably due to the mobility hindrance of the PEEK chains with the addition of GNP. This behaviour was previously reported with the addition of nano-SiO_2_ particles, short glass fibres, or Gd_2_O_3_ [3,37,42].

PEEK/GNP (10 wt %) crystallized from glass sample has not been included in this model, owing to be crystallized partially during quenching (Table 1).

**Figure 5:**
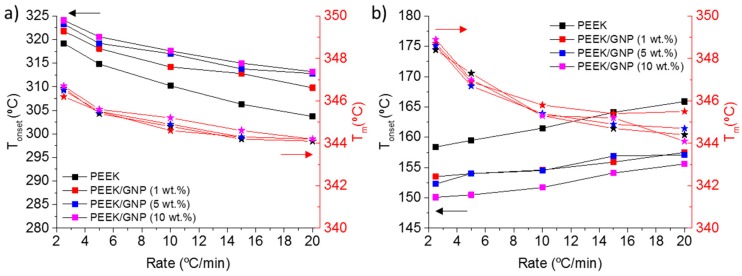
Crystallization temperature onset (square symbols) and melting temperature (star symbols) against cooling (**a**) and heating rate (**b**).

In addition, these experiments allow for analysing the influence of cooling or heating rates in the crystallization from melt or from glass, respectively. Figure 5 shows the beginning of crystallization, *T*_onset_, and melting temperature, *T*_m_, at different cooling and heating rates. As it was expected in the crystallization from melt, higher melting temperatures were observed for lower cooling rate, because the crystallization was at higher temperature, and then a thicker crystal layer was formed [1,11]. The effect of GNP was similar at all cooling rates, showing parallel trends, such in *T*_onset_ as *T*_m_, in samples with and without GNP. In the case of samples that were crystallized from glass, lower heating rate provided crystallization at lower temperatures, nevertheless given higher *T*_m_, probably due to the recrystallization processes at high temperatures favoured by the slower heating rates. Finally, the GNP content produced cold crystallization at lower temperature independent on heating rate, this systematic effect was also observed for the melting temperatures, except for composite with 10 wt % in GNP. This experiment was not a pure crystallization from glass, because it was partially crystallized during the cooling, as it was mentioned previously and it will be discussed in the next section.

### 3.3. Non-Isothermal Crystallization Morphology

**Figure 6:**
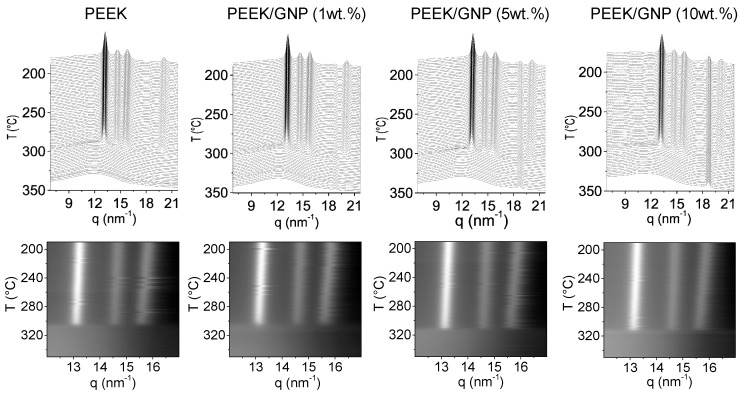
Simultaneous time-resolved wide angle synchrotron X-ray radiation (WAXS) patterns (upper) and intensity profiles of three main PEEK diffractions (down) for crystallization from melt of PEEK and PEEK/GNP nanocomposites.

In this section, both crystallizations from melt and from glass state were performed at 10 °C/min by X-ray Synchrotron radiation. Figure 6 shows the time-resolved WAXS during a cooling experiment from melt (upper) and the intensity profiles of the PEEK diffractions (down). The three main characteristic diffraction peaks for orthorhombic phase (110), (111) and (200) were clearly observed in all samples [43,44,45], with similar scattering vector (q) positions at 13.24, 14.66, and 15.81 nm^−1^, respectively, and also a similar intensity ratio among them. The difference was found in the crystallization temperature, being higher with the GNP content, which is in line with DSC results that were previously described. The crystallization process was similar in all samples, the three main diffraction peaks appeared simultaneously, and their evolution with the temperatures was equivalent in all cases, as intensity profiles showed. On the other hand, the peak around 18.8 nm^−1^ related as GNP peak had higher intensity when the major percentages of GNP were added, and no variation in this diffraction was observed during the cooling.

**Figure 7:**
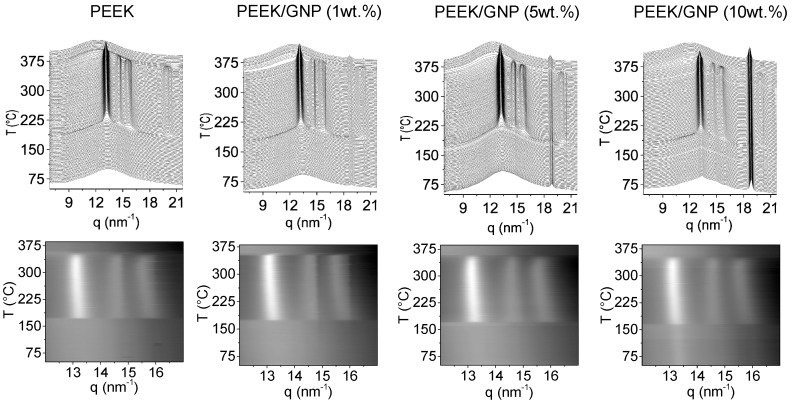
Simultaneous time-resolved WAXS patterns (**upper**) and intensity profiles of three main PEEK diffractions (**down**) for crystallization from glass (cold crystallization) for PEEK and PEEK/GNP nanocomposites.

Figure 7 shows the heating of the quenched samples from room temperature. At low temperature, pure PEEK and composites with 1 and 5 wt % of GNP showed a typical amorphous broad diffraction peak, confirming the amorphous glass state that was also observed by DSC. However, in nanocomposite with 10 wt % of GNP, three emerging diffraction peaks were already observed and located in the expected q for orthorhombic crystal phase [6]. This fact indicates that this composite was partially crystallized during fast cooling, supporting DSC results that are summarized in Table 1. After *T*_g_, cold crystallization was observed in all samples. Again, the three main diffractions of PEEK appeared at the same time, but in this case at lower temperature according the GNP content. Likewise, no variation was observed for GNP diffraction peak at 18.8 nm^−1^. The ratio among peak diffraction intensities as well as the variation of the peak positions during the whole heating was similar in all samples, as was observed in the intensities profiles of Figure 7. Therefore, GNP did not affect the crystal morphology of PEEK either in the cooling from melt or in heating from the glass state.

**Figure 8:**
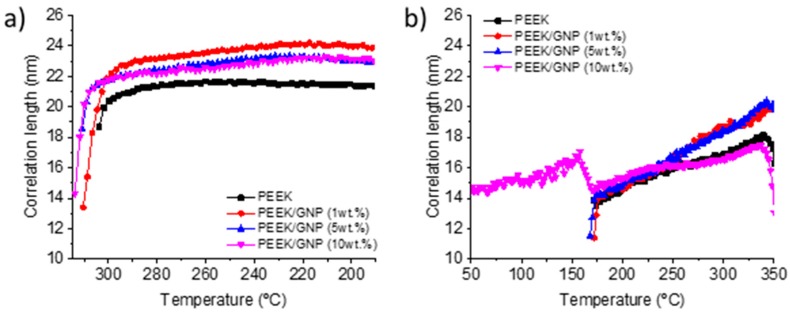
Temperature dependence, on cooling from melt (**a**) and heating from glass (**b**) of the correlation length of (110) reflection peak for all samples.

On the other hand, when comparing semicrystalline patterns after cold crystallization and crystallization from melt, the lower crystallinity that was obtained by DSC results (Table 1) was corroborated. The three main reflections after cold crystallization were much broader, as Figure 7 shows, and the intensities ratio of reflections 200 and 111 against 110 were lower as well, demonstrating that the crystals formed during cold crystallization were smaller and worse crystalline structures than crystals formed during melt crystallization. This is normal when taking into account the much lower crystallization temperature during cold crystallization processes. Although, the GNP did not affect the morphology and the evolution of the crystal phase during both non-isothermal crystallizations. It was noted that the correlation length calculated by Scherrer equation (K∙2π/w_(110)_, being w_(110)_ the width at half-height of the 110 reflection peak in *q* (scattering vector), and considering values of 1 for K (shape factor)) [46], was higher for nanocomposite materials. These correlation lengths could be related to the production of better crystalline ordering for nanocomposites. In Figure 8, the longest correlation length is observed for nanocomposite with 1 wt % after its crystallization from melt. Higher GNP content produced lower correlation length, owing to crystallization at slightly higher temperature. The higher viscosity, which reduced the polymer mobility, might justify this effect. For this reason, during the heating of amorphous samples, the variation of the width between the cold crystallization and final melting was more extent. Though, the cold crystallization began at lower temperature, the lower mobility of chain produced worse crystalline ordering, this effect was reverted with the temperature increase, the polymer chain acquired more mobility and the nucleating effect improved the ordering, yielding higher correlation lengths for composites. Nanocomposite with 10 wt % of GNP content did not follow this behaviour, because its cold crystallization was affected by the pristine crystal that formed during the fast cooling (quenching process).

**Figure 9:**
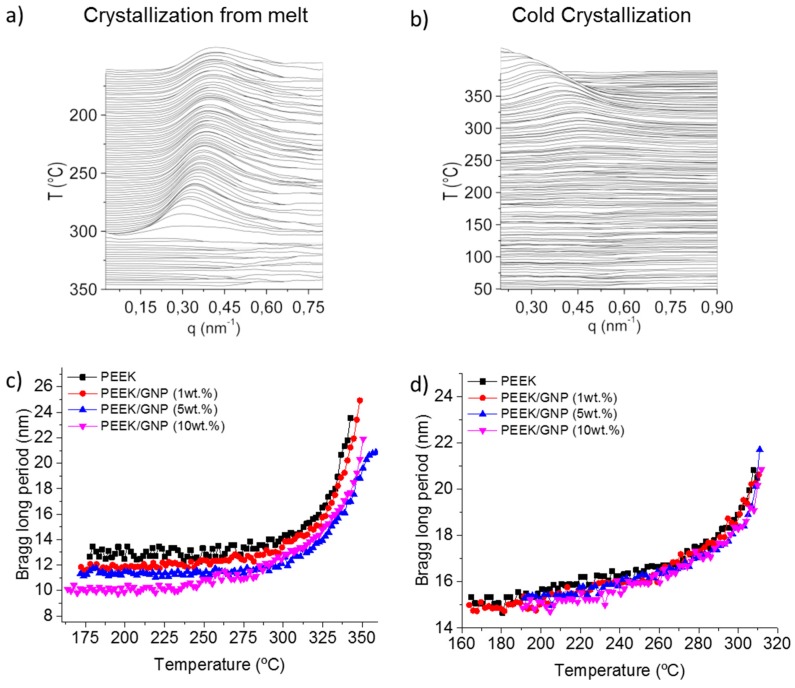
Small angle synchrotron X-ray radiation (SAXS) synchrotron profiles corresponding for pure PEEK samples, crystallization from melt (**a**), crystallization from glass (**b**). Temperature dependence, on Bragg long period for samples crystallized during cooling from melt (**c**), and during heating from glass phase (**d**).

Finally, the evolution of the crystal lamella thicknesses could be analysed by SAXS. Figure 9a,b shows the SAXS profile for pure PEEK samples after Lorentz correction from melt and from glass crystallization, respectively. Figure S8 shows these profiles for composites that were crystallized from melt and from glass. All of the samples presented a clear long periods (L) which are also compared in Figure 9c,d. It is well known that crystallization of PEEK is developed in two steps, this phenomena produces a reduction of the L along the crystallization process [47]. During the primary crystallization, the homogenous molten state produces a thicker lamella. The subsequent secondary crystallization, in the restrained melt inside of primary spherulites, produces a thinner lamellar layer. In our case, GNP affected the primary crystallization because it caused heterogeneous nucleation, and it might also affect the secondary crystallization because this one was at lower temperature, where the polymer mobility was more restricted. Figure 9c shows the variation of L during the cooling, where two clearly stages were observed. At high temperatures, mainly at a temperature range corresponding to exothermic peak by DSC, L decreased fast in all samples. This sharp drop was associated mainly with the primary crystallization. Later, L decreased low and continuously, and this thinning was associated to the secondary crystallization [7]. Such nanocomposites as pure PEEK presented the same L profile without variation in the transition between both stages. The main difference was found in the L values, because, though the crystallization began earlier due to nucleating effect, the lamella thickness was reduced with the GNP content. This effect was even more marked during the cold crystallization, where L was reduced from 13 to 10 nm with the GNP content. This L reduction was associated to the higher nucleation density due to the heterogeneous nucleation of the graphene, as it has been observed in graphene nanocomposite of polypropylene [48] and polyethylene [49]. Cold crystallization happened at lower temperature than crystallization from melt. Subsequently, more intense differences in the nucleation density between nanocomposite and pure matrix were expected, therefore the thinning effect in L was more pronounced, as it was observed in Figure 9d.

## 4. Conclusions

The effect of the graphene nanoplatelets in the non-isothermal crystallization of PEEK matrices of concentration from 1 to 10 wt % has been evaluated. Firstly, the mobility of the polymer chain was restricted in the presence of GNP, as their rheological behaviour revealed. Concentration of 1 wt % only showed the beginning of rubbery plateau formation at low frequencies, but kept the Newtonian behaviour. G′ at high frequency was practically unaffected due to a strong shear thinning behaviour. However, higher concentration of GNP produced a clear increase in complex viscosity, G′, and G″ in the whole frequency range analysed, besides the vanishing of Newtonian behaviour due to the interactions particle-particle that dominated over the polymer-filler interactions, forming an incipient interconnected network of GNP.

GNP had two main effects in the PEEK crystallization process. First, a nucleating effect that was observed by the increase of the beginning of the crystallization (*T*_onset_) in the nanocomposites during the crystallization from melt, and the lower cold crystallization temperature (*T*_cc_) during the crystallization from glass. Second, hindered crystallization processes because the crystallization times were increased with the addition of GNP, indicating that polymer chains had more constrains to crystallize, as it was expected after rheological measurements. The Avrami analysis by Jeziorny that was done for the primary crystallization indicated that the nucleation and growth of non-isothermal crystallization from melt and cold crystallization are different. The addition of GNP reduced the Avrami exponent only in the cold crystallization. The reduced of the PEEK mobility by the GNP affected the values of the *Z*_c_, crystallization rate, showing a downward trend with the GNP content.

Real time WAXS/SAXS X-ray measurements during both non-isothermal crystallization processes showed that GNP did not affect the orthorhombic phase of PEEK and the evolution of the crystal phase during the crystallization processes. However, the correlation length of the crystal was higher for nanocomposites materials in both non-isothermal crystallization processes. The highest correlation lengths were found in 1 wt % GNP composite, due to the best combination of higher nucleation temperature and not so restricted polymer mobility when compared to 5 and 10 wt % GNP composites. Finally, the thinner long periods (L) that were observed for nanocomposites confirmed the higher nucleation density due to the heterogeneous nucleation of the GNP; this effect was more pronounced in cold crystallization process due to the lower temperature in which this processes occurred.

In resume, GNP favours the crystallization rate because the nucleation occurs at higher temperature. GNP also reduces the polymer chain mobility reducing the crystallization rate, but the influence of this effect can be reduced in crystallization at higher temperatures. Therefore, an isothermal crystallization study combining rheological measurements and isothermal crystallization followed by DSC and X-ray synchrotron radiation will be our next goal. In processing point of view, the nucleant effect of GNP seems to have more influence than the reduction of polymer chain mobility, because nanocomposites with high concentration of GNP cannot be quenched in complete amorphous glass during a fast cooling.

## Figures and Tables

**Table 1:** Temperature at the beginning of crystallization (*T*_onset_), crystallization temperature (*T*_c_), full width at half maximum value (FWHM) crystallization times calculated from *T*_onset_ to *T*_offset_ (*t*_c_), melting enthalpy (Δ*H*_m_), degree of crystallization (*X*_c_), and melting temperature (*T*_m_) during non-isothermal crystallization from melt and glass state.

Crystallization	Parameters	PEEK	1 wt % GNP	5 wt % GNP	10 wt % GNP
**From melt (−10 °C/min)**	***T*_onset_ (°C)**	310.2	315.9	317	317.6
	***T*_c_ (°C)**	302.4	306.7	306.9	308.1
	**FWHM (min)**	0.49	0.60	0.85	0.80
	***t*_c_ (min)**	1.3	1.71	1.98	1.96
	**Δ*H*_m_ (J/g)**	52.2	50.6	48.4	46.5
	***X*_c_ (%)**	40.1	39.3	39.2	39.7
	***T*_m_ (°C)**	344.8	344.6	344.9	345.2
**From glass (10 °C/min)**	***T*_onset_ (°C)**	161.5	154.6	154.5	151.7
	***T*_cc_ (°C)**	169.3	165.3	164.8	163
	**FWHM (min)**	0.32	0.43	0.41	0.66
	***t*_c_ (min)**	1.07	1.38	1.32	1.48
	**Δ*H*_m_ (J/g)**	40.65	39.9	40.1	34.02
	***X*_cc_ (%)**	31.3	31.0	32.5	29.1
	**Δ*H*_m_ − Δ*H*_cc_ (J/g)**	0.7	0.9	0.5	15
	***T*_m_ (°C)**	345.3	345.8	345.4	345.3

**Table 2:** Calculated n and *Z*_c_ values at different cooling (negative rates) and heating rates (positive rates), calculated from the slopes and intercepts of the linear parts of the modified Avrami plots.

β (°C/min)	PEEK		1 wt % GNP		5 wt % GNP		10 wt % GNP	
	n	*Z*_c_	n	Z_c_	n	*Z*_c_	n	*Z*_c_
−2.5	2.4	0.49	2.36	0.42	2.24	0.40	2.37	0.38
−5	2.39	0.90	2.25	0.85	2.3	0.79	2.3	0.85
−10	2.25	1.09	2.38	1.06	2.22	1.02	2.5	0.97
−15	2.14	1.10	2.41	1.08	2.23	1.06	2.59	1.07
−20	2.15	1.10	2.34	1.08	2.3	1.06	2.63	1.08
2.5	3.5	0.36	2.84	0.18	3.2	0.11		
5	3.4	0.87	2.38	0.68	2.81	0.70		
10	3.72	1.05	2.36	0.95	2.48	0.97		
15	4.19	1.17	2.78	1.01	2.6	1.07		
20	3.86	1.18	2.73	1.08	2.36	1.07		

## Supplementary Materials

The supplementary materials are available online at http://www.mdpi.com/2073-4360/11/1/124/s1.
