# Supplementary material for: Non-Isothermal Crystallization Behavior of PEEK/Graphene Nanoplatelets Composites from Melt and Glass States
Source: Polymers (Basel). 2019 Jan 12;11(1):124. doi: 10.3390/polym11010124 (PMC6401876; doi:10.3390/polym11010124)
Supplement: Supplementary file 1 [file polymers-11-00124-s001.pdf]

Supplementary Materials:

Non-isothermal crystallization behavior of PEEK/Graphene nanoplatelets composites from melt and glass states

Ángel Alvaredo[1], María Isabel Martín[2], Pere Castell[3], Roberto Guzmán de Villoria[1,2], Juan P. Fernández-Blázquez[1,*]

[1] IMDEA Materials Institute, C/ Eric Kandel 2, 28906 Getafe, Madrid, Spain

[2] FIDAMC, Foundation for the Research, Development and Application of Composite Materials, Avda. Rita Levi Montalcini 29, Tecnogetafe, 28906 Getafe, Madrid, Spain

[3] Fundación AITIIP, Pol. Ind. Empresarium, C/ Romero 12, 50720 Zaragoza, Spain

* Correspondence: juanpedro.fernandez@imdea.org; Tel.: +34 91 549 34 22

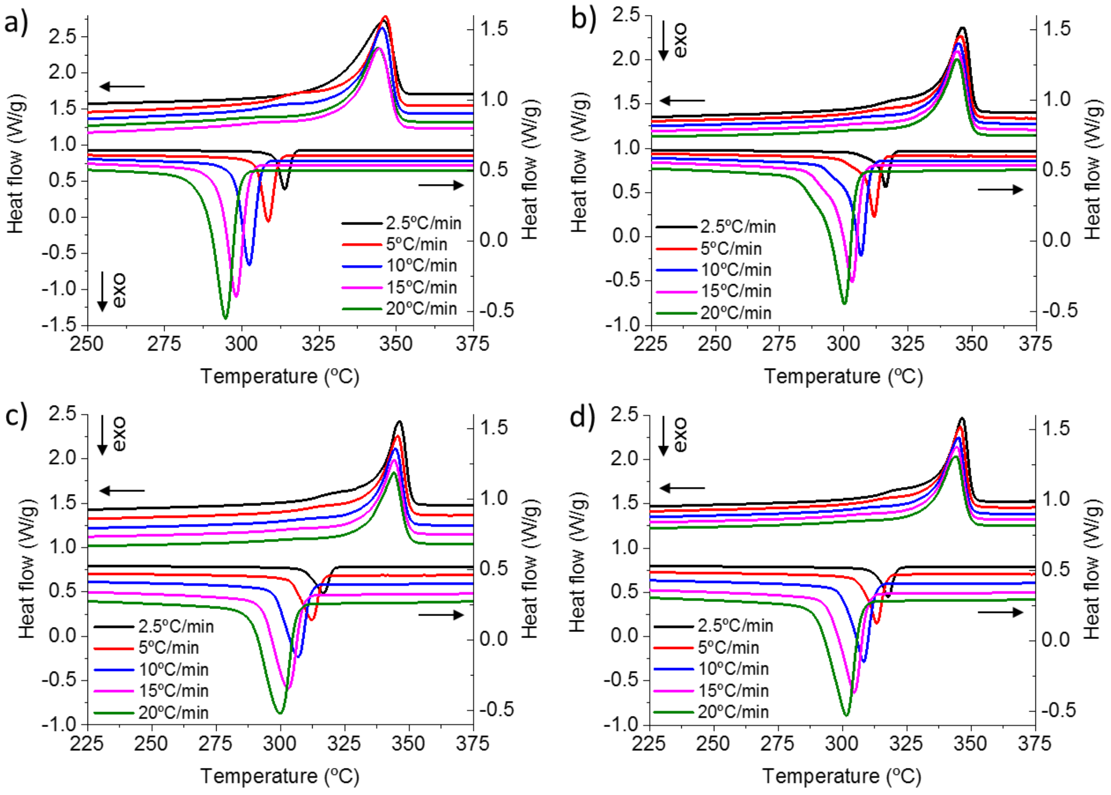

Figure S1. DSC thermographs during heating and cooling of a) neat PEEK, b) PEEK/GNP (1wt.%), c) PEEK/GNP (5wt.%) and d) PEEK/GNP (10wt.%).

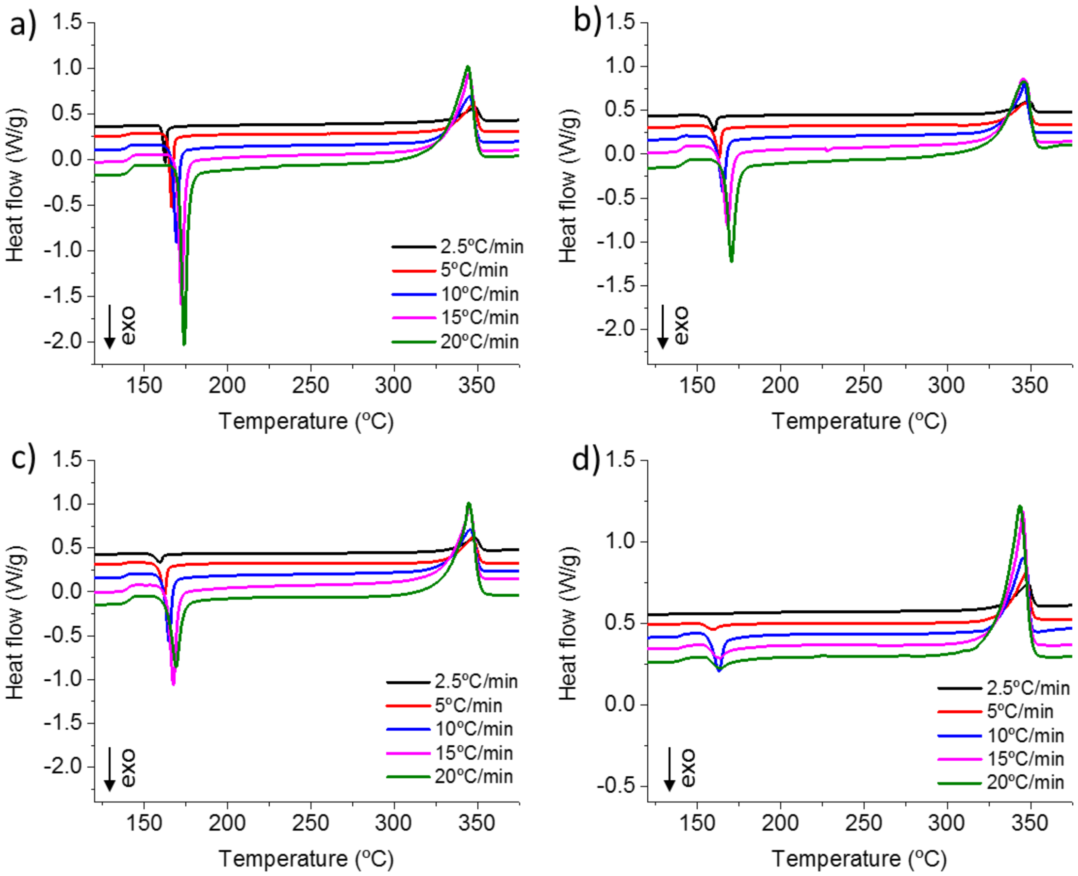

Figure S2. DSC thermographs during heating of amorphous samples a) neat PEEK, b) PEEK/GNP (1wt.%), c) PEEK/GNP (5wt.%) and d) PEEK/GNP (10wt.%).

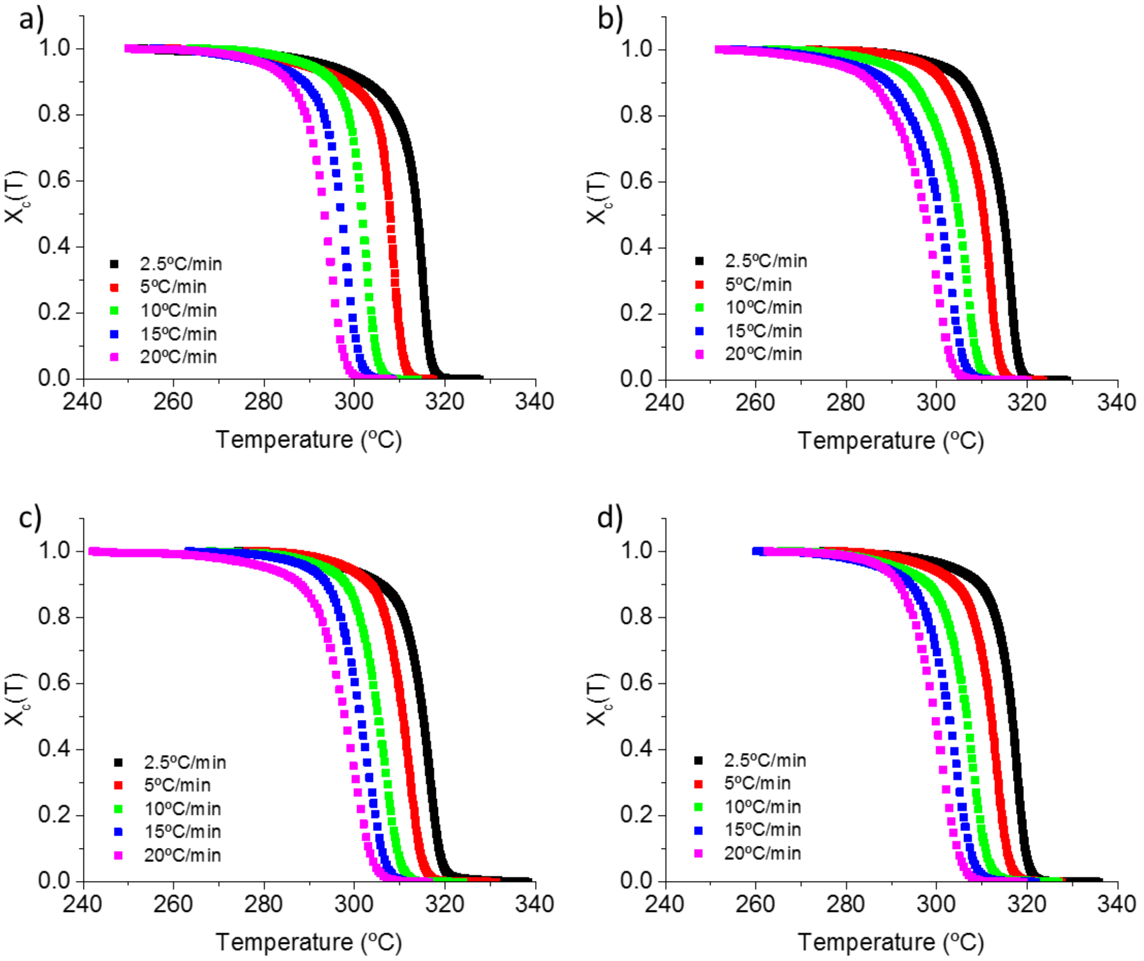

Figure S3. Relative crystallinity against temperature for all samples: a) neat PEEK, b) PEEK/GNP (1wt.%), c) PEEK/GNP (5wt.%) and d) PEEK/GNP (10wt.%).

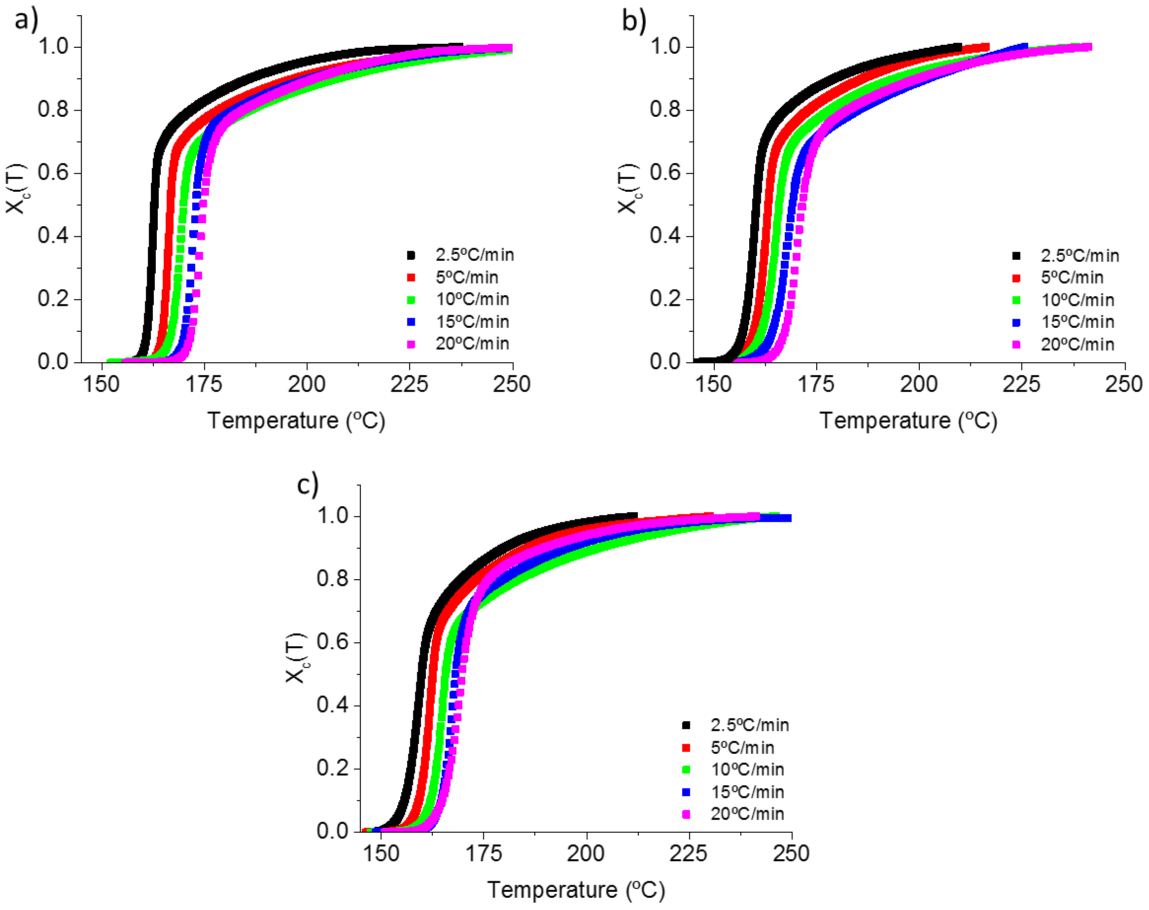

Figure S4. Relative crystallinity against temperature for all the samples crystallized from melt: a) neat PEEK, b) PEEK/GNP (1wt.%) and c) PEEK/GNP (5wt.%).

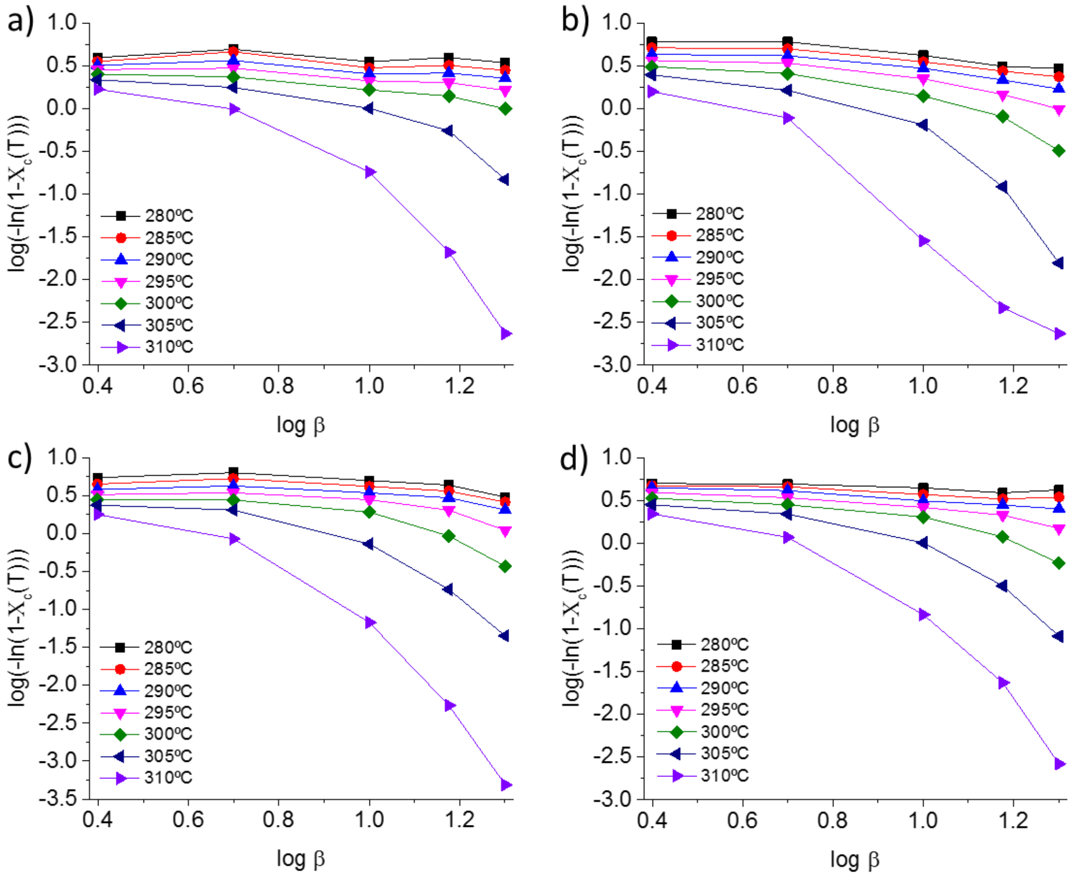

Figure S5. Ozawa plots of log (-ln (1-XC (T))) against log β for a) neat PEEK b) PEEK/GNP (1wt.%), c) PEEK/GNP (5wt.%) and d) PEEK/GNP (10wt.%) samples crystallized from melt.

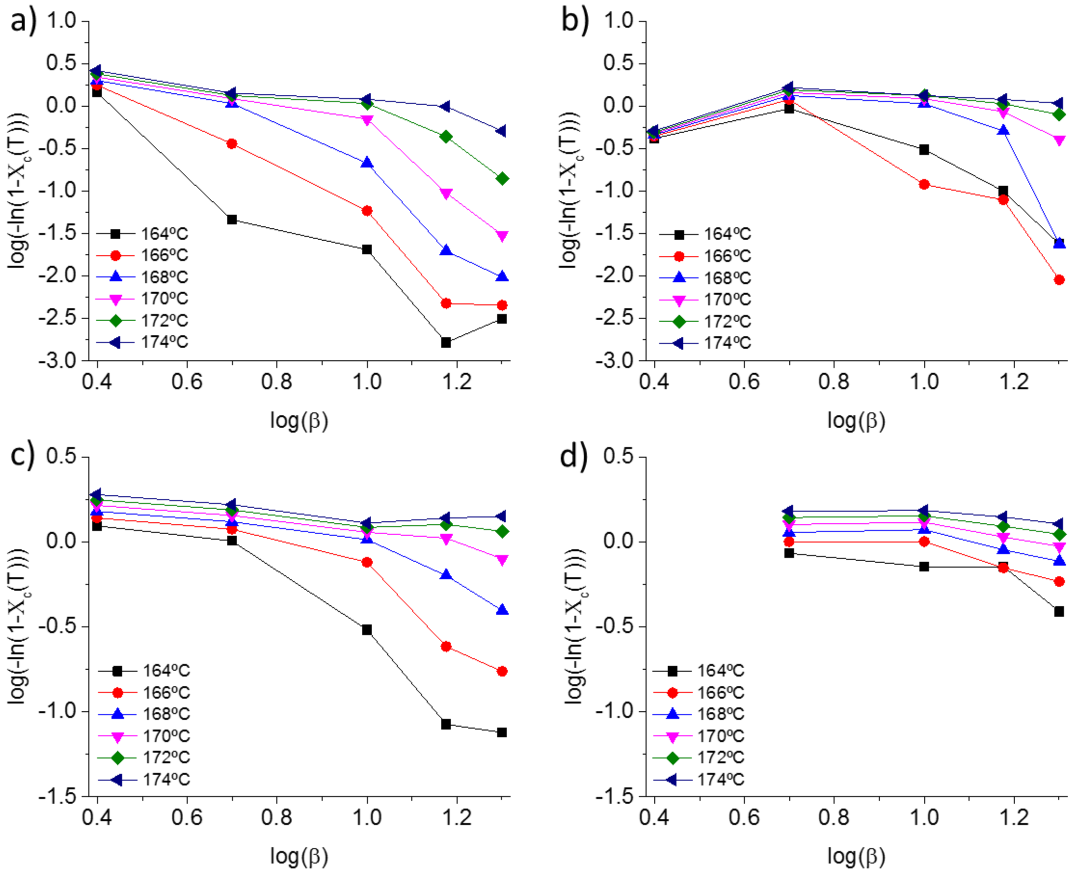

Figure S6. Ozawa plots of log (-ln (1-XC (T))) against log β for a) neat PEEK b) PEEK/GNP (1wt.%), c) PEEK/GNP (5wt.%) and d) PEEK/GNP (10wt.%) samples crystallized from glass.

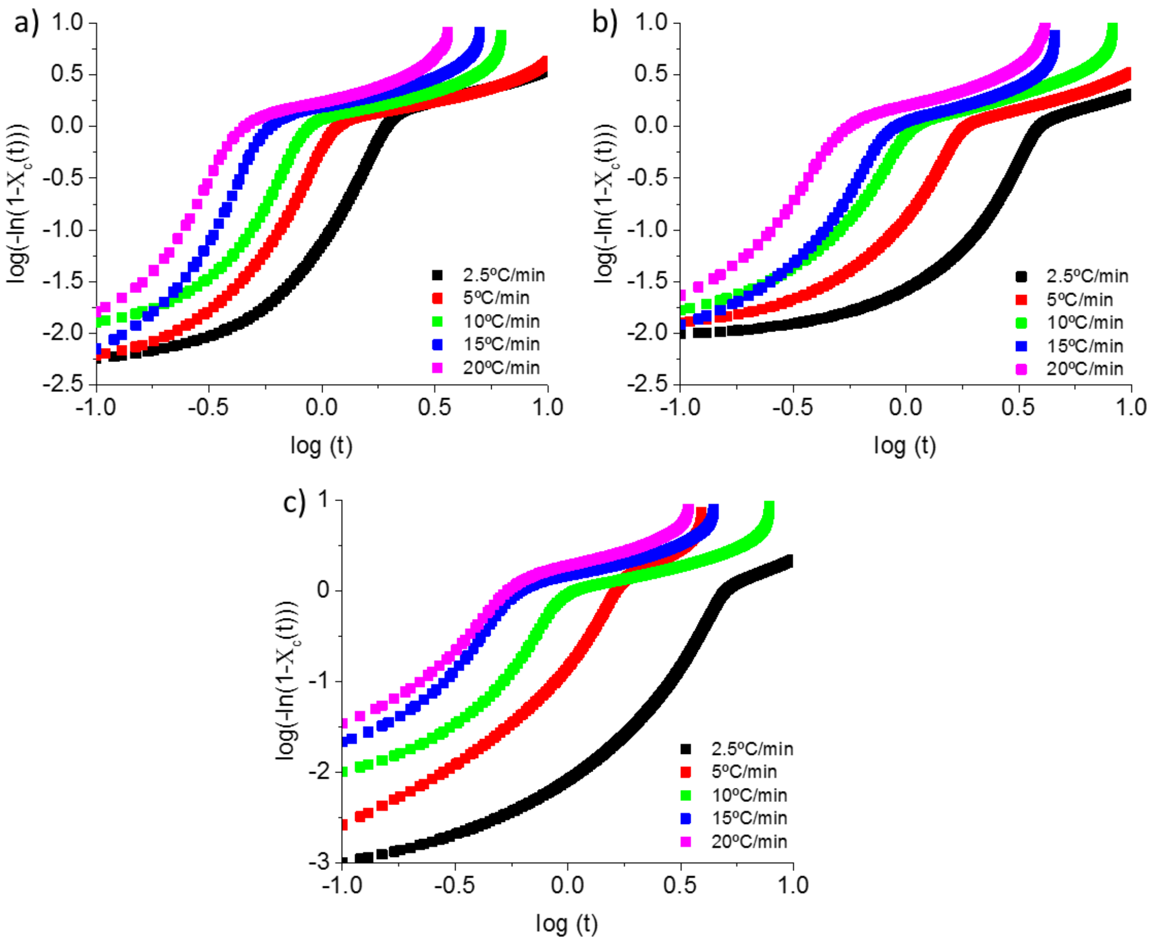

*Figure S7***.** Modified Avrami plots at various heating rates.a) neat PEEK, b) PEEK/GNP (1wt.%) and c) PEEK/GNP (5wt.%).

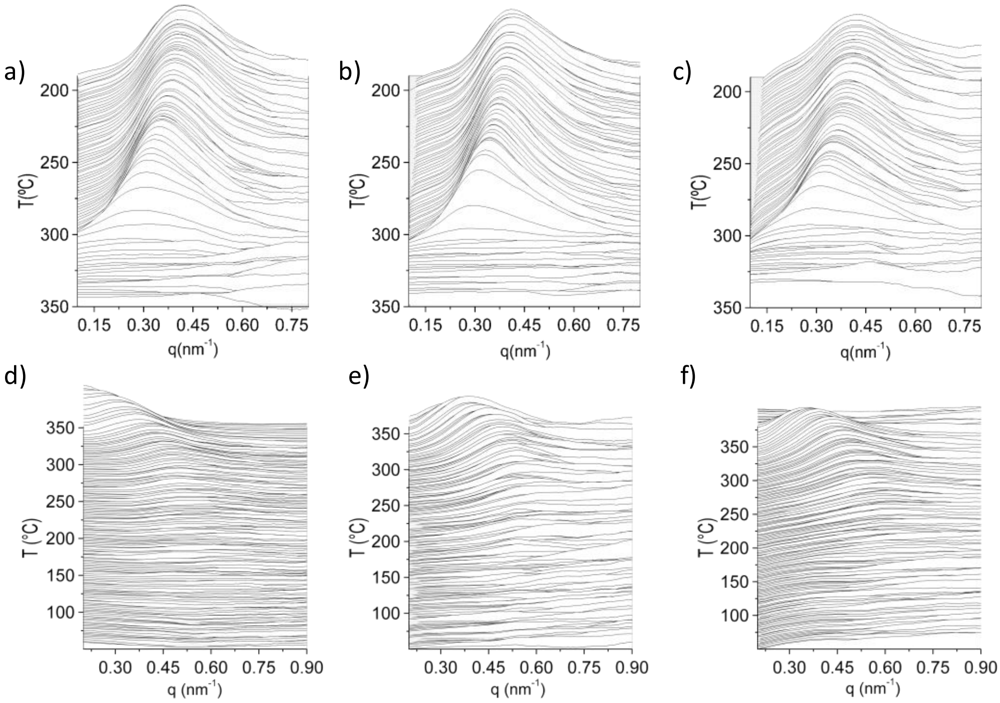

***Figure S*8.** SAXS synchrotron profiles corresponding for a) PEEK/GNP (1wt.%), b) PEEK/GNP (5wt.%), and c) PEEK/GNP (10wt.%) samples crystallized from melt. Samples crystallized from glass: d) PEEK/GNP (1wt.%), e) PEEK/GNP (5wt.%), and f) PEEK/GNP (10wt.%).
